# Association between mean platelet volume and severity of rheumatoid arthritis

**DOI:** 10.11604/pamj.2017.27.276.12228

**Published:** 2017-08-14

**Authors:** Jamileh Moghimi, Farahnaz Ghahremanfard, Maryam Salari, Raheb Ghorbani

**Affiliations:** 1 Social Determinants of Health Research Center, Faculty of Medicine, Semnan University of Medical Sciences, Semnan, Iran; 2 Internal Medicine Department, Semnan University of Medical Sciences, Semnan, Iran

**Keywords:** Mean platelet volume, rheumatoid arthritis, disease activity score

## Abstract

**Introduction:**

In recent years, it is suggested that platelet histogram indices, such as mean platelet volume (MPV) may be related to the activity of rheumatoid arthritis (RA). The aim of this study was to assess relationship between MPV and activity of rheumatoid arthritis.

**Methods:**

Sixty consecutive patients fulfilling the American College of Rheumatology (ACR) criteria for RA were recruited from the rheumatology outpatient clinics in Semnan, Iran. Current disease activity score (DAS-28 score) was assessed at baseline, 2 months and 4 months after the admission time and beginning of the treatment schedule. Complete blood count (including MPV), C-reactive protein (CRP) and ESR were measured in each visit. MPV was analyzed by the Cell Dyne 3500 automated blood cell counter.

**Results:**

There was a significant reduction in DAS-28 score within 4 months of total assessment (from 4.47 ± 2.24 versus 3.18 ± 1.55) (p < 0.001). There was no significant difference in MPV levels at the three study time points. No significant correlations were observed between the DAS-28 score and mean MPV levels at the same time points. The rate of positive CRP was decreased within the same period (p = 0.002); however, the trend of the changes in other laboratory parameters including MPV, platelet count and ESR values was not significant. The measurement of MPV value did not correlate with disease activity in RA patients within 4 months of treatment scheduling.

**Conclusion:**

Although therapeutic regimens, which improve RA manifestations, can reduce RA activity, they had no effect on MPV during this time period. It seems MPV may not be able to predict disease activity in RA patients.

## Introduction

Currently, many physicians are interested in platelet indices, particularly the platelet volume, because it may reflect the platelet function better than the platelet count itself. Nowadays, platelet volume is usually reported in routine complete blood count results. The mean platelet volume (MPV) reflects the platelet size. Elevation of MPV is a suggestive paraclinical indicator for platelet production and activation [1]. Several studies showed the role of platelet indices such as MPV in thrombosis, immunity, inflammation and angiogenesis [2, 3]. In clinical settings, changes in this simple marker are linked to cardiovascular disease, brain stroke risk profile, dyslipidemia, hypertension, non-insulin dependent diabetes mellitus, overweight [4–7]. Disease-specific and cardiovascular confounding factors affect the direction of MPV changes. Platelet count estimations complicate the interpretation of MPV values in thrombocytopenia. It would seem that the size of circulating platelets is dependent on the intensity of systemic inflammation, with contrasting features of MPV in high and low-grade inflammatory disorders and the course of anti-inflammatory treatment [3]. Rheumatoid arthritis (RA) is a chronic inflammatory disease which characterized by tenderness, swelling and stiffness of the joints, with progressive destruction of cartilage and bone [8]. It has been recently identified that MPV is one of the most widely used surrogate markers of platelet function and has been shown to reflect inflammatory burden and disease activity in rheumatic arthritis. Thus, serial measurements of MPV may be worthwhile in prediction of developing of different diseases [9]. The aim of the current study was to assess relationship between the changes of MPV and indices of RA activity.

## Methods

**Participants:** Sixty consecutive patients, who met the 2010 rheumatoid arthritis classification criteria [10] were recruited from our rheumatology outpatient clinics from 21 march 2015 to 19 march 2016. Patients were predominantly female.

**Inclusion criteria:** Non-smoker patients without known established cardiovascular disease, diabetes mellitus, hypertension, other autoimmune diseases included the study.

**Measurements**: Patients underwent thorough clinical and laboratory evaluation, including complete medical history and current disease activity score (DAS-28) [11], at baseline, 2 months and 4 months after the admission time and beginning of the treatment schedule. Blood samples (6 ml) were collected between 8-11 a.m. Venipuncture was performed using 23G needle and blood samples were collected first in vacutainers for C-reactive protein(CRP) and Erythrocyte sedimentation rate (ESR) measurements and then in EDTA-containing tubes for hematology tests. Hematological analyses were performed rapidly, according to the previously published recommendations to avoid swelling of platelets stored in EDTA-containing test tubes [12]. MPV was analyzed by the Cell Dyne 3500 automated blood cell counter. The size of platelets was quantified by flow cytometry on a cell-by-cell basis by measuring the intensity of scattered light at angular range of 2°-3°. MPV measurements were obtained from the platelet volume histograms. All patients were on different dosages of hydroxychloroquin, methotrexate and prednisolone. None of them was on anti-tumor necrosis factor (anti-TNF) or other biologic agents.

**Ethical considerations**: This study was approved by the local research ethics committee of the Semnan University of Medical Sciences and all participants provided written informed consent.

**Statistical analysis**: Results were presented as Mean, Standard Deviation (SD), Median and Interquartile Range (IQR) for quantitative variables and were summarized by absolute frequencies and percentages for categorical variables. Statistical analysis was performed by SPSS 18.00 Software, using Kolmogorov-Smirnov test, to test the normality of data, Mann-Whitney test to compare differences between two independent and Friedman test, for testing the difference between 3 related samples, when the dependent variable is not normally distributed. Also we used Spearman's rho Correlation Coefficient to calculate correlation of two ordinal variables. P-value less than 0.05 considered statistically significant.

## Results

**Figure 1:**
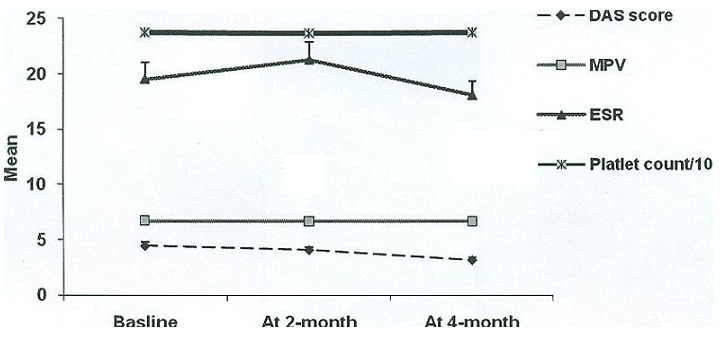
Trend in the changes of study parameters within the study time period

Mean ± SD of age was 49.5 ± 12.3 years (ranged 25 to 74 years) and 51 (85.0%) were female. The Mean ± SD duration of disease was 6.2 ± 6.5 years that was statistically similar in men and women (7.8 ± 9.7 years versus 5.9 ± 5.8 years, p = 0.786). At baseline, mean DAS-28 score was 4.47 ± 2.24. As showed in Table 1 and regarding changes in study parameters within 4 months of the assessment, significant difference was noted in the rate of disease activity that significantly reduced at 4-month time point. The trend of the changes in DAS-28 score was significant, so that it was significantly decreased from its baseline level of 4.47 ± 2.24 to 3.18 ± 1.55 over 4 months of total assessment (Figure 1 and Table 1). Comparing mean MPV values between the groups showed no significant difference in MPV levels at the three study time points. No significant correlations were observed between the DAS-28 score and mean MPV levels at the same time points (Table 2). The rate of positive CRP was decreased within the same period (p = 0.002); however, the trend of the changes in other laboratory parameters including PLT and ESR values was not significant (Table 1).

**Table 1:** The changes in study parameters within baseline, 2 and 4 months after treatment

Parameter	At baseline				At 2-month				At 4-month				p-value
	Mean	SD^a^	Median	IQR^b^	Mean	SD	Median	IQR	Mean	SD	Median	IQR	
DAS score	4.47	2.24	4.15	4.00	4.10	1.91	4.00	2.82	3.18	1.55	2.65	1.65	<0.001
MPV	6.75	2.08	6.80	3.77	6.66	2.15	6.30	4.05	6.65	2.19	6.40	4.25	0.983
Platelet count	237.2	63.0	240.5	89.2	236.4	68.2	233.0	93.7	237.6	60.2	242.5	79.2	0.239
ESR	19.5	11.7	17.5	19.7	21.8	12.2	20.0	17.0	18.1	9.7	18.0	15.0	0.025
CRP	n		%		n		%		n		%		0.002
0	32		53.3		36		60.0		46		76.7		
1	14		23.3		12		20.0		8		13.3		
2	9		15.0		6		10.0		4		6.7		
3	4		6.7		6		10.0		2		3.3		
4	1		1.7		0		0.0		0		0.0		

a Standard Deviation(SD)

b Interquartile Range(IQR)

**Table 2:** correlation between mean platelet volume (MPV) and disease activity score (DAS) within the study time period

Time period	r	p-value
At baseline	0.050	0.706
At 2-month	-0.107	0.415
At 4-month	-0.133	0.310

## Discussion

In present study, regarding trend of the changes in RA severity, DAS-28 score was gradually decreased within 4 months of study implementation and changes in MPV and other biomarkers were not significant within the study period. In recent years, it is suggested that Platelet histogram indices such as MPV may be linked to the activity of RA. Rapid control of disease activity in RA patients at least during several months has substantially optimized. There are some conflicting data in the literature concerning the values of MPV in patients with RA [13]. Yazici S et al, studied 97 patients with control group and showed that MPV correlated with inflammatory markers (ESR & CRP) and disease activity (DAS-28 score) in RA patients.They reported higher values of MPV in patients with RA, which were correlated with the DAS-28 disease activity score, decreasing after the treatment [14]. In another study Gasparyan et al compare data, were obtained from 400 RA patients and 360 non-RA controls from the local population. They found significantly increased MPV in RA patients compared with controls (P = 0.001). In RA patients, blood pressure greater than 140/90 mmHg was associated with high levels of MPV. They recommended prospective studies to explore the role of MPV as a marker for cardiovascular risk in RA [15]. High-grade inflammation accompanies a decrease of MPV in RA, possibly due to the increased consumption of platelets at the sites of rheumatoid inflammation. A reverse shift of MPV results from the suppression of inflammation by disease-modifying and anti-TNF-alpha agents. The results of the study expand perspectives on the use of MPV in conditions associated with high-grade inflammation, particularly RA, for monitoring anti-inflammatory treatment [16]. Gasparyan et al again mentioned more prospective studies with large numbers of patients to ascertain associations of high and low values of MPV with diverse markers of inflammation and vascular pathology [17]. Some studies showed that RA patients with high disease activity tend to have smaller size of platelets than those at remission. Kisacik et al found small size of platelets in RA patients at active stage, compared to the same parameter after a 2-month conventional anti-rheumatic treatment. They reported lower values of MPV in patients with active RA than controls and these values increased significantly after treatment, but remained lower than in control patients [16].

The best reasons of controversy among studies are described by Gasparian et al; as they mention in their article: although the regulation of platelet function and aging, is dependent on the ploidy (ability to reduplicate DNA) and maturity of thrombopoietic progenitors, different cytokines & factors in circulation affect on the platelet production such as: Interleukin-1 (IL-1);Interleukin-6 (IL-6); Tumor necrosis factor-a(TNF-a) and platelet activation in different physiologic and pathologic situation result in time dependent change of platelet indices. On other hand, MPV is measured by cell counters using impedance and optical effects. The discordance between the results of different and even the same cell counters limits the interchangeable use of MPV. This can explain, at least partly, why hematological. Laboratories sometimes do not display the MPV and some other indices of platelet function [3]. Otherwise, previous studies reported the fact that the MPV is highly dependent on the time of storage until the analysis [14]. Inaccurate measurement of platelet indices may be due to inappropriate blood sampling and storing. The platelet indices have been shown to be sensitive to the differences in blood sample anticoagulation, storage temperature and delays in processing. It is widely accepted that platelet swelling in test tubes can be minimized by rapid processing of samples (within less than 1h) or by using tubes with sodium citrate (time-dependent changes are more characteristic for EDTA). Notably, within the first hour of sampling, MPV values of EDTA samples are at least 9% higher than those of citrated samples. Earlier, it was suggested using tubes with high concentration of sodium citrate to obtain more reliable measures of MPV. The lack of agreement may reflect differences in the assessed pathways of platelet activation, poor laboratory standardization as well as diurnal variation of MPV in both physiological and pathological conditions [17]. One of the limitations of this study was the small sample size. More prospective studies with large numbers of patients recommend ascertaining associations of consecutive measurements of MPV with RA disease activity, especially with considering different effective factors on MPV value.

## Conclusion

Although therapeutic regimens, which improve RA manifestations, can reduce RA activity, they had no effect on MPV during this time period. It seems MPV may not be able to predict disease activity in RA patients within 4 months of treatment scheduling

### What is known about this topic

- In recent years, it is suggested that Platelet histogram indices such as MPV may be linked to the activity of RA.

### What this study adds

- MPV value may not be able to predict diseases activity in RA patients.

## Competing interests

The authors declare no competing interest.
